# Special Issue on HOX Genes in Development

**DOI:** 10.3390/jdb5020005

**Published:** 2017-05-10

**Authors:** Vincenzo Zappavigna

**Affiliations:** Department of Life Sciences, University of Modena and Reggio Emilia, Via G. Campi 213/d, Modena 41125, Italy; zappavigna.vincenzo@unimore.it; Tel.: +39-0592055537

This Special Issue of *Journal of Developmental Biology* (*JDB*) covers an indeed very “special” (at least to me) family of highly evolutionarily conserved genes, the *Hox* genes. Despite over three decades having passed since the discovery of the homeobox, the excellent level and the wide range of topics of the articles and reviews published in this Special Issue testify the long-standing and ongoing interest in the functions of this unique gene family. The studies gathered in this issue of *JDB* cover subjects ranging from the use of *Hox* genes as a paradigm for the development of computational methods of protein family classification, to the role of *Hox* genes in the development and evolution of appendices, and the mechanisms underlying the expression of specific Hox proteins.

Limb development has traditionally represented a paradigm for the study of pattern and organ formation in vertebrates. For a long time, *Hox* genes have been recognized to play crucial roles in limb development. Two different reviews in this issue are focused on the role of *Hox* genes in limb development, one from the perspective of limb field formation, the other touching upon the broader issue of their role in appendage evolution. How the limb-forming fields within the lateral plate mesoderm were acquired during evolution represents one of the most intriguing questions of the evo–devo field. Tanaka [1] elegantly summarizes what is known regarding the possible role of *Hox* genes in the generation of limb-forming fields, bringing together comparative gene expression analyses and gene manipulation results to reconstruct, from cephalochordates and agnates to land vertebrates, the sequential events leading to limb-field formation. Leite-Castro et al. [2] review the role of *HoxA* genes in another hot topic of evo–devo: the fin to limb transition. More specifically, they focus on the modifications in the protein sequence and gene expression regulatory mechanisms of two key *Hox* genes, *Hoxa11* and *Hoxa13*, which are highly indicted of having promoted appendage evolution.

How *Hox* gene products, especially those of paralogous *Hox* genes, gain their functional specificity in vivo is also a long-standing question in the field. Zandvakili and Gebelein [3] review recent advances on this issue, especially taking into consideration the interactions between Hox proteins and partner proteins, such as the PBC (Pbx, ceh-20, exd domain proteins) and the HMP (Homothorax, Meis, Prep), the role of the chromatin context at target regulatory regions, and that of post-translational modifications capable of influencing Hox DNA binding properties.

The increasing recognition of the implication of *Hox* gene misregulation in human disease is also covered by two reviews that discuss the role of *Hox* genes in cardiovascular pathologies and of *HOXA5* in leukemias, in breast, colorectal, and non-small cell lung cancers. More specifically, Roux and Zaffran [4] describe the emerging role of anterior *Hox* genes in the development of the cardiovascular system and highlight their possible implication in congenital heart defects. While Jeannotte et al. [5] review, in depth, what is known about the functions of the *Hoxa5* gene, starting from the information gained by analyzing its knockout mouse model, and discussing its pleiotropy, and the broad range of actions of *Hoxa5* during development.

Finally, two papers address the classification and evolutionary relationship of organisms and of proteins, using *Hox* gene number, cluster organization, and sequence divergences as a measure of evolutionary diversification. The comparative analysis of *Hox* gene expression patterns and cluster composition in different animal groups is essential to understand the mechanisms by which body plan modifications evolved, leading to radiation. Barucca et al. [6] review data regarding the number of *Hox* genes and cluster structure in the clade of *Lophotrochozoa*, whose phyla display a wide variety of morphological diversities. Collinearity of expression and *Hox* cluster structure are discussed in relation to the evolutionary relationships between the various lophotrochozoan phyla. While Hueber and Frickney [7] report a new computational method, based on pairwise sequence comparisons, for solving the problems that conventional phylogenetic methods encounter when dealing with protein sequences that are either highly or only slightly divergent. They use Hox and Para-Hox proteins as a paradigm to demonstrate the soundness of their method, providing an improved classification and phylogenetic reconstruction of the relationships between Hox and Para-Hox proteins.

In conclusion, the variety and the excellent level of the papers published in this Special Issue are tangible proof that most, if not all, of the open questions regarding the role of *Hox* genes in developmental and disease processes are still arousing interest and inspiration for active investigation.
